# A social VR-based collaborative exergame for rehabilitation: codesign, development and user study

**DOI:** 10.1007/s10055-022-00721-8

**Published:** 2022-11-28

**Authors:** Syed Hammad Hussain Shah, Anniken Susanne T. Karlsen, Mads Solberg, Ibrahim A. Hameed

**Affiliations:** 1 Department of ICT and Natural Sciences, Faculty of Information Technology and Electrical Engineering, Norwegian University of Science and Technology (NTNU), Larsgårdsvegen 2, 6009 Ålesund, Norway; 2 Department of Health Sciences, Faculty of Medicine and Health Science, Norwegian University of Science and Technology (NTNU), Larsgårdsvegen 2, 6009 Ålesund, Norway

**Keywords:** Immersive healthcare, Virtual reality, Social interaction, Metaverse, Exergaming, User-centered design, Eldercare, Motivation, Physical rehabilitation

## Abstract

Immersive virtual reality (VR)-based exercise video games (exergames) are increasingly being employed as a supportive intervention in rehabilitation programs to promote engagement in physical activity, especially for elderly users. A multifaceted and iterative codesign process is essential to develop sustainable exergaming solutions. The social aspect is considered one of the key motivating factors in exergames; however, research on the social aspect of VR exergames has been limited. Previous studies have relied on competitiveness in exergames, but research has shown that competition can lead to adverse effects on users. With the aim of motivating elderly individuals to participate in physical exercise and improving social connectedness during rehabilitation, this work presents a social VR-based collaborative exergame codesigned with elderly participants and therapists. This exergame stimulates full-body exercise and supports social collaboration among users through a collaborative game task. Furthermore, this article presents a user study based on a mixed-methods approach to gather user feedback on exergame design and the effect of social collaboration versus playing alone in a VR exergame in terms of physical exertion and motivation. This study spanned five weeks (99 exergaming sessions) with 14 elderly participants divided into two groups, one playing collaboratively and the other playing individually. Between-group comparisons were performed at baseline (first week) and in the fourth week, and within-group comparisons were performed in the fifth week, when the participants played the exergame in counterbalanced order. In contrast to the first week, the participants exergaming collaboratively in the fourth week reported significantly higher intrinsic motivation on all subscales (enjoyment: *p* < 0.02, effort: *p* < 0.002, usefulness: *p* < 0.01) and physical exertion (*p* < 0.001) than those playing alone. Thereafter, exergaming in counterbalanced order during the fifth week resulted in significant differences (medium to large effect size) within groups. The participants found the social VR gameplay enjoyable and agreed that collaboration played a vital role in their motivation. They reported various health benefits, a minimal increase in symptoms of simulator sickness, and excellent usability scores (83.75±13.3). In this work, we also identify various key design principles to support healthcare professionals, researchers and industrial experts in developing ergonomic and sustainable VR-based exergames for senior citizens.

## Introduction

Physical inactivity is common among elderly people (Jaarsma et al. 2015; Goršič et al. 2017). Due to the lack of motivation, many find it challenging to attain and sustain minimally recommended levels of physical activity over time (Jones et al. 2020). This is unfortunate since regular activity is highly recommended by health professionals, as it is beneficial for both physical and cognitive health (Van Santen et al. 2018; Cacciata et al. 2019; Wu et al. 2019). Additionally, research has suggested that motivated individuals are more likely to follow exercise regimens prescribed by therapists (Maclean et al. 2000). Regarding motivation, self-determination theory describes it as a continuum of individual dispositions from amotivation via extrinsic motivation to self-determination, i.e., intrinsic motivation, whereby action is taken due to perceived interest, enjoyment and self-satisfaction (Ryan and Deci 2020). Accordingly, exercise motivation can be improved by increasing individuals’ perceptions of enjoyment and usefulness. Other theories foreground social connectedness as an important motivational factor for performing physical activity (Maslow 1981; Cacioppo and Cacioppo 2014; Ryan and Deci 2020). Social connectedness with relatives, friends, and even health professionals can be an important source of motivation for elderly people to be more active (Peiris et al. 2012). For this age group, social connectedness via group exercise (Mehra et al. 2016; Najafabadi et al. 2019) supports both exercise adherence and performance (Hamari and Koivisto 2015).

Various assistive technologies have been utilized for physical training and rehabilitation purposes in a variety of settings. As the number of elderly people is growing, there is a need to facilitate and support active and healthy aging, with exergames potentially supporting this goal (Wu et al. 2015; Nagano et al. 2016; WHO 2021). As early as 20 years ago, commercial video game consoles, such as Nintendo, Wii Fit and Xbox Kinect, allowed players to interact with a virtual gaming environment using full-body movements to support real-world physical exercises (Cao et al. 2021). Similar approaches can be used to promote physical exercise among the elderly (Skjæret et al. 2016), and exergames can motivate and enable elderly users to participate in enjoyable as well as physically strenuous games (Lyons et al. 2012). Multiple reviews have surveyed how exergames can support exercise adherence and motivation among elderly people, potentially leading to improved quality of life (Bruun-Pedersen et al. 2016; Kappen et al. 2019; Reis et al. 2019).

Most currently available applications have investigated the potential of off-the-shelf exergames, based on commercial platforms, in a range of domains (Nawaz et al. 2016; Júnior et al. 2021). One lesson from these interventions is that positive health outcomes must be founded primarily on behavioral and social interventions rather than technological innovations alone (Høeg et al. 2019; Schroeder 2007). Exploring user preferences and motivational factors related to exergames through user-centered design methods is therefore imperative and of no less value than technical innovations (Skjæret et al. 2016). Many studies on exergames have based experiences for physical training and exercise on 2D interfaces (Xiao et al. 2017; Bezerra et al. 2018; Fraser et al. 2018; Campo-Prieto et al. 2021). Conversely, exergames that explore opportunities in immersive virtual reality (VR) remain understudied (Campo-Prieto et al. 2021). Nevertheless, interest in VR-based physical training for elderly users has been increasing (Tieri et al. 2018; Campo-Prieto et al. 2021). A VR-based exergame immerses users in a three-dimensional virtual environment through a head-mounted display. Full-body movements in physical space correspond with movements in virtual space, and vice versa. Most of these VR applications have been designed as single-player games, either for acquiring motor skills (Levac et al. 2019) or for repetitive exercise (Bruun-Pedersen et al. 2016; Stamm et al. 2022). A VR-system for strength training of young people, based on the Oculus Rift head-mounted display and the Kinect V2-sensor, has been demonstrated in previous research (Tanaka and Hirakawa 2016). While the study reviewed the design process and gameplay, it was not clear from the results whether the system was effective in encouraging users to engage in physical activity. Moreover, a VR-based exertion game based on a similar configuration has been developed for physically rehabilitation (Eckert et al. 2017). This game appeared to improve movement, but it was not clear that it led to improvements in exercise motivation and routines. To boost players’ performance in VR-based exergames, an interactive feedforward mechanism was proposed to support self-competition, whereby the individual competes against its own past performance (Barathi et al. 2018). According to this study, the interactive feedforward method was beneficial for the players’ intrinsic motivation. In another recent study (Babadi and Daneshmandi 2021), VR-based balance training was found enjoyable and effective compared to conventional methods. A recent review (Piech and Czernicki 2021) also suggests that VR-based rehabilitation may complement conventional methods of physiotherapy.

More recently, the ubiquity of social isolation among vulnerable elderly people during the ongoing COVID-19 pandemic has also accentuated the potential value of social VR. In social VR, users communicate through embodied avatars as digital representations of themselves, controlling these avatars via physical embodied movements. Large commercial players, such as ‘Meta’ (formerly ‘Facebook’) and Microsoft, now consider virtual worlds to be the next frontier, foreshadowing huge investments in the social VR also called ‘Metaverse’ (Microsoft 2021a; Meta 2022). Some exergaming applications have also integrated social aspects. Nevertheless, a recent review of exergames for users above the age of 50 found few publications explicitly focusing on the role of social dimensions; thus, scholars have called for more research in this domain (Marker and Staiano 2015; Nguyen et al. 2017). One line of investigation explored the motivational role of peer socialization in exergames through competitive or collaborative play (Gorsic and Novak 2016; Meekes and Stanmore 2017). A recent study (Høeg et al. 2021) reported on a cross-sectional study of elderly people collaborating with close acquaintances in a virtual bicycling exercise. While the players found the experience enjoyable and captivating, even with minimal social interaction through embodied avatars, this study did not investigate their motivation as a consequence of repeated use, and the results may have been confounded by the novelty effect. Furthermore, an exploratory study (Shah et al. 2022) was conducted recently to evaluate the general discomfort and experience of elderly individuals pertaining to their interaction with immersive virtual world and communication with each other in social VR. The results were promising in terms of usability, general experience and interest in social VR. A longitudinal study (Júnior et al. 2021) found significant improvements in adherence and functional capacity among elderly users of a 2D screen-based exergame who competed with peers compared to those who played alone. Like many other social exergames, the game in this study relied on adversarial game mechanics instead of centering the game around social collaboration. A recent pilot study on play mode effects of exergaming on depression in older adults (Li et al. 2020) found that team-play could potentially reduce the experience of loneliness. Competition-based games have been shown to reduce intrinsic motivation (Song et al. 2013) and lead to increased stress (Goršič et al. 2017) and aggression (Pereira et al. 2019). Competition can also be challenging for individuals undergoing rehabilitation. Limited evidence of how elderly users experience social VR-based rehabilitation through full-body exercise and avatar-mediated social interaction in VR (Baker et al. 2021), as well as the possible negative repercussions of competitiveness in exergames, supports the necessity of further research into the effects of social collaboration in VR-based exergames for rehabilitation.

### Aim of the present study

In this study, we aimed to evaluate the potential of social collaboration-based VR exergames to influence social experiences and exercise motivation among elderly users. We introduced a social VR-based collaborative exergame that enabled elderly participants to participate in avatar-mediated social interaction in a distributed immersive virtual space and play a collaborative exergame based on a joint game task that stimulated physical exercise. The exergame centered around functional, full-body movements. Our first objective was to codesign and develop this exergame with elderly users and physiotherapists, thereby tailoring the game design to the needs of elderly users for an effective, ergonomic, enjoyable, and safe user experience. Based on this codesign process, we also wanted to identify important game design principles and key insights that could be beneficial for others in designing VR-based exergames for this user group. Thereafter, we conducted a five-week study based on repeated play of the exergame to mitigate the novelty effect that can result from a mere demonstration (Høeg et al. 2021). We collected data using a mixed-methods approach with different outcome measures to evaluate the exergame design in terms of user experience and compare the results between those who played collaboratively (PC) and those who played alone (PA). The primary objective was to investigate the participants’ perception and the usability of this exergame, gather self-reports about perceived health benefits from the participants and their therapists, and address the following research questions.

- How does social collaboration in the VR-based exergame influence participants’ motivation, enjoyment and effort compared to playing alone over time?
- How does social collaboration in the VR-based exergame influence participants’ exercise performance (physical exertion) compared to playing alone over time?
- How does social collaboration in the VR-based exergame influence participants’ gaming experience compared to playing alone?

## Methods

This study introduced a social VR-based exergame to facilitate full-body exercise through a fruit-picking game and social collaboration through team-based game tasks and reward mechanisms. The choice of the game and its overall design were decided through the user-centered design process. The fundamental exergame design was a two-player fruit-picking challenge that placed players together in front of each other at a table decorated with multiple fruit baskets in VR. The challenge was to collect the maximum number of fruits in a defined period and was set as a team goal instead of an individual goal. The exergame automatically configured itself for the placement of game objects according to the player’s physique. It provided an opportunity for the users to share the immersive virtual environment using the internet from any location. The exergame architecture was designed in such a way that games for more than two players could easily be integrated into the existing application through minimal changes in the game design. A detailed discussion of the codesign and development of this exergame is presented in Sect. 2.2.

### Participants

To conduct our study, we collaborated with physiotherapists at a municipal rehabilitation center in Western Norway. The physiotherapists contacted potential participants based on the following selection criteria. The participants had to be above 60 years of age. Candidates included those who had visited, were currently residing or were awaiting an appointment at the municipal rehabilitation center. Eligible candidates should have experienced some reduction in their physical strength due to physical inactivity but be able to stand for up to 15 minutes without assistive devices. Exclusion criteria included cognitive impairments, visual impairments or weak eyesight, and issues that would prevent candidates from participating in the intervention. A screening process reduced the short list from 45 potential candidates to 14 participants. It was not feasible to recruit a larger sample due to practical constraints during COVID-19. The study was recommended by the Data Protection Official at the Norwegian Centre for Research Data (ref.: 508625). Participation was based on informed consent, per the Norwegian Personal Data Act.

**Fig. 1:**
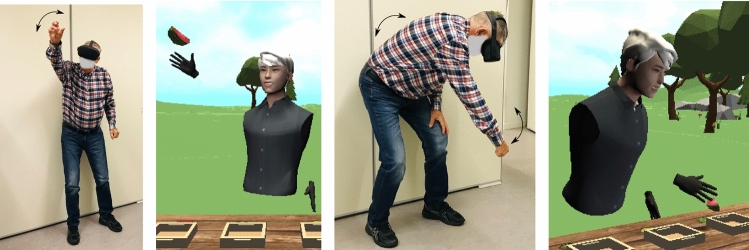
Diagrams showing trunk, arm, leg, wrist, hand and body movements in correspondence with the gameplay activity

### Exergame codesign and development

In this section, we describe our codesign process, which was conducted with the elderly participants and physiotherapists to design and develop the exergame. This process was initiated via conversations with the physiotherapists. These professionals highlighted the need for additional ways to keep elderly patients engaged in physical activities throughout the rehabilitation process. In their experience, many elderly clients (both outpatients and those residing at the care facility) lack motivation to participate in physical training and as a result often fail to follow their prescribed rehabilitation programs or to fulfill the minimum requirements for routine exercise. Another concern was the COVID-19 situation, which made it risky to organize group exercise. Hence, the physiotherapists saw a need to find ways to integrate enjoyable and social activities into their programs, and an exergame informed by therapeutic goals could support this effort. As a result of this contextual analysis, we initiated a codesign and development process (which took place over six months) to develop an ergonomically designed VR-based exergame to achieve this goal.

#### Phase I: Therapeutic goals

In this phase, the initial codesign and development occurred over four meetings with the physiotherapists to discuss further details of the study and showcase the affordances of VR technology. The central task was identifying training exercises that could meet the relevant therapeutic goals and were safe for elderly people to perform in VR. Based on input from the physiotherapists about suitable full-body exercises, along with a literature review, we decided that the exergame should support training for balance, mobility, and range of motion in different body parts. A decision was made to implement a cross-training exercise that included posture transitions to induce the player to perform cervical movements (flexion, extension and rotation), shoulder movements (arms raised towards the front and side), elbow and wrist movements, trunk movements (flexion, extension, and rotation in backbone and hips), and leg movements (stepping toward the front, back and sides). The posture transitions included in the exercise are shown in Fig. 1.

#### Phase II: Brainstorming about the exergame design

In the second phase, members of the team involved elderly participants at the rehabilitation center in codesigning the game. After giving informed consent, they were introduced to the purpose of the study, VR technology and the exercises suggested by the physiotherapists. Two meetings were conducted to brainstorm about what type of game the elderly participants would like to play. A priority was to integrate the mechanics of movement into a game design where the player moves her body according to posture transitions as per the recommended exercises. Another important consideration was to design the game tasks in a way that supported social collaboration. This process addressed five design elements: environment design, interface design, interaction design, mechanics of movements, and reward mechanisms. The participants were given multiple options for the game type and shown videos of different games. Based on inputs from the physiotherapists and the elderly participants, we decided to keep the flow and game story simple. After deliberation, an object-picking game was selected as the initial prototype because it satisfied the requirements for movement mechanics and simplicity. Moreover, the elderly users preferred the virtual milieu to represent the natural environment.

**Fig. 2:**
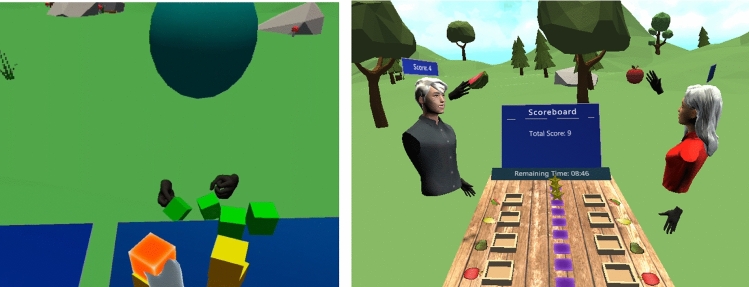
Evolution of exergame design during the co-design and development process

#### Phase III: Initial prototype development and testing

Having defined these parameters and empathized and ideated with our codesigners through the second phase, we developed an exergame prototype in which the player had to pick cube-shaped interactive objects. The immersive landscape was composed of grass, trees, flowers, and rocks, with chirping birds and ambient wind sound, as shown in Fig. 2. The design of the virtual environment was motivated by the discussions in Phase I and reports documenting a positive reception by senior citizens of nature-based immersive virtual environments (Chan et al. 2021). We also implemented a configuration process to control the instantiation and placement of interactive objects in 3D space according to the posture transitions shown in Fig. 1. The aim was to induce the players to perform body movements conforming to the exercises identified in the first phase. Based on this configuration, objects were randomly placed at one of the top-right, top-left, bottom-right, and bottom-left corner positions, calculated with respect to the head position of the player in the virtual environment. The exergame placed interactive objects according to the player’s height on runtime to make the exergame adaptive. Both the vertical and horizontal distance between the head position and the four corner positions was set at 0.5 meters, approximating the average arm length of an adult. A table set at a height of 0.5 meters was placed in front of the players for placement of the cube-shaped interactive objects that the players were supposed to pick. This table height was set slightly lower than that of a normal table (0.7–0.8 m), to induce the players to stimulate trunk movements.

A virtual 3D keyboard was provided to the players to enter their name, select a gender, and choose a gaming mode (PA or PC). Embodied virtual hands controlled either via wireless hand controllers attached to a VR headset or direct hand manipulation through hand tracking were provided for virtual interactions, such as using the keyboard and grabbing, touching, or hovering over game objects. The embodied avatar, representing the user in VR, was a 3D sphere with virtual hands collocated with the player’s orientation. The players could communicate via representative avatars and voice communication. After developing the initial prototype, we tested it with the physiotherapists and elderly participants. No reward mechanism was implemented at this stage.

**Fig. 3:**
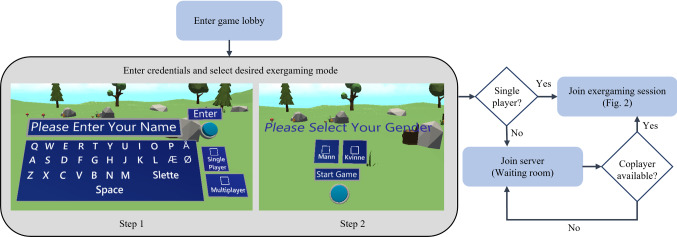
User flow from initiation to joining an exergame session

#### Phase IV: Brainstorming and revising the exergame design

This phase began with engaging the codesigners in discussions about necessary changes in the prototype to adapt it more appropriately to elderly players. According to the physiotherapists, the movement mechanics were aligned with the posture transitions in the recommended exercise. The elderly participants also appreciated the user experience for the proposed interface, as well as the system response, and the practical details of initiating the exergame application. Central to the interaction design was the fact that the elderly players preferred to use direct hand manipulation to interact with the virtual game objects due to ease of use, comfort, and the lower effort needed to remember the controls. The participants also suggested redesigning the game objects, such as the table and cube-shaped objects, to be more similar to real-world objects. Therefore, we accommodated a wooden table and four different fruits. In addition to a configuration process for placement of the interactive fruits, we designed the game so that the objects approached the players from the front, instead of placing them in a static position, to create a more engaging experience. Regarding the avatar design, the participants reported that it was strange to interact with a sphere-shaped avatar. We therefore designed and integrated human avatars for both genders. The humanoid avatar was assigned to players based on their selected gender. We also discussed potential reward mechanisms and the duration of each session with the participants. We placed four baskets (one dedicated to each fruit type) on the virtual tabletop. The reward mechanism was set up in such a way that putting fruit in the correct basket would yield one point, and there was no penalty for putting fruit in the wrong basket. We also included star tokens as a virtual reward and badges to increase players’ motivation. The baskets were positioned in such a way that fruits instantiated on the right side had to be put into the baskets on the player’s left side and vice versa. The goal was to induce players to move their arms diagonally and to stimulate rotation in the back and hips as well as to shift their weight for balance training. We then designed a collaboration mode among the coplayers through a reward mechanism relying on the collective effort instead of individual efforts. A player could choose to play alone or with a coplayer, and the duration of each game session in both gaming modes was initially set to 10 minutes and then, increased to 15 minutes depending upon the player’s performance. Information about the time remaining and a virtual scoreboard were also provided.

#### Phase V: Retesting the exergame

After completing another iteration that implemented changes based on the recommendations from the participants, we conducted another user testing session. Here, the participants approved the overall game design except for two game features. In the original version, the virtual objects approached the player from the front. However, the players found it too challenging to interact with these moving objects as the pace increased throughout the game. We therefore implemented a solution whereby the objects were positioned statically. Moreover, it was difficult for the elderly players to perceive the depth of objects in the virtual environment. To improve these perceptions, we assigned different colors to highlight game objects when interacting to touch and grab them and different sound effects to inform the player about the completion of an action, such as pressing a button, grabbing or releasing an object, or earning a game point. At this stage, hand tracking and a function to save the tracked data of hands’ positions to measure the level of physical exertion by the participants were also implemented. Having integrated these changes, we went through another testing phase with the exergame. At this stage, the design was finalized by the participants and physiotherapists. The game evolution during the codesign process is shown in Fig. 2. The final version could be played in either a standing or a sitting position depending upon the user due to the game design being adaptive. Fig. 3 shows the user flow from initiating to joining an exergaming session.

### Materials and apparatus

The exergame was developed in Unity3D, version 2019.4.15f1. Currently, the game supports a VR headset named Meta Quest (formerly Oculus Quest) (Oculus 2021). The virtual environment was created using off-the-shelf assets of Unity3D and the Simple-Nature-Pack asset tool (JustCreate 2021). The Mixed Reality Toolkit (MRTK) version 2.5.1 (Microsoft 2021b) was used to create the user interface comprising various controls, such as buttons, checkboxes, and texts. Moreover, the MRTK was used to integrate the interaction mechanisms with user interface controls, such as pressing a button, touching or grabbing objects with one or both hands, and hovering over objects. The use of direct hand manipulation was made possible through the Oculus Integration Software Development Kit (SDK) (Oculus 2020) for hand tracking along with MRTK. Multiplayer options supporting a distributed connection over the wireless network between game players and networked information about avatar embodiment, voice communication and shared game objects were implemented using Normcore SDK (Normal-Inc. 2020).

### Outcome measures

#### Demographic questionnaire

A demographic questionnaire was developed to collect background information about the participants. It included variables like gender, age, medication use, physical health issues, whether they were working or retired, computer skills, familiarity with electronic devices, and experience with VR.

#### System usability scale

The system usability scale (SUS) is a Likert scale-based instrument with 10 items to assess the usability of a system (Brooke 1996). Meldrum et al. found the SUS to be a reliable measurement for assistive technologies in clinical settings (Meldrum et al. 2012). The SUS contains different questions related to efficiency, effectiveness, and satisfaction regarding the system under observation. These questions are rated based on a scale ranging from one (“strongly disagree”) to five (“strongly agree”) (Brooke 1996).

#### Simulator sickness questionnaire

The simulator sickness questionnaire (SSQ) (Kennedy et al. 1993) measures the extremity of simulator sickness. It is a brief measurement tool containing 16 questions. Although the SSQ was initially designed to gauge motion sickness in simulators for aviation and military uses, it is popular for assessing the negative effects of VR technology. The user is asked to rate symptoms related to oculomotor disturbances (O), nausea (N) and disorientation (D) experienced in VR systems. The scale for rating symptoms ranges from none (0) to slight (1), moderate (2), and severe (3) (Kennedy et al. 1993). Symptoms charted with the SSQ include general discomfort, fatigue, headache, eyestrain, difficulty focusing, increased salivation, sweating, nausea, difficulty concentrating, blurred vision, dizziness with eyes open, dizziness with eyes closed, vertigo and stomach awareness. Here, the SSQ was used to evaluate simulator sickness before and after the user’s exposure. The total score (TS) and aggregate of subfactors (O, N, D) were calculated for preexposure, postexposure and change in score. When the total score is below five, the symptoms are considered minor, and a simulator with a score above 20 is considered poor.

#### Game experience questionnaire

The game experience questionnaire (GEQ)(Poels et al. 2007) was used to assess players’ experiences during and after the gaming session. We adopted two modules from the GEQ. The in-game module had seven submodules comprising 42 items, measured on a Likert scale ranging from not at all (0) to extremely (4). The submodules were sensory and imaginative immersion, tension, competence, flow, negative affect, positive affect, and challenge, each with six items. The postgame module had four submodules comprising 17 items in total. The submodules were negative experiences (six items), positive experiences (six items), tiredness (two items) and returning to reality (three items).

#### Motivation

The intrinsic motivation inventory (IMI) has been used to measure the motivation of participants involved in various studies (Ryan and Deci 2000). The IMI is a multidimensional measurement grounded on self-determination theory and focused on assessing subjective experiences. It was designed for use after experimental interventions, to measure the subject’s impressions of a task. The questionnaire has four subscales comprising 32 items. The subscales were interest/enjoyment (seven items), effort/importance (five items), value/usefulness (seven items), relatedness (eight items) and pressure/tension (five items). The first four subscales were utilized in this study. Responses to each statement were recorded on a seven-point scale ranging from “not at all true” (1) to “partially true” (4) and “ very true” (7). Both positive and negative statements were present in each subscale to avoid the memorability effect. The score for each negative statement was calculated by subtracting its score from eight to convert it to a positive statement in order to calculate the total score.

#### Virtual embodiment questionnaire

Roth et al. (Roth and Latoschik 2020) designed a virtual embodiment questionnaire (VEQ) to assess feelings of embodiment. It is based on three dimensions: ownership (acceptance and ownership of the human-like virtual body), agency (sense of control over movements in the virtual body) and change (perception of change in the body, such as weight, appearance and size) (Roth and Latoschik 2020). Overall, the VEQ comprised 12 items and used a Likert scale ranging from strongly disagree (1) to strongly agree (7).

#### Qualitative feedback

Qualitative data were collected through collaborative meetings, field observations, interviews and a questionnaire based on text input with the physiotherapists and elderly participants during the codesign process and five-week testing. During the latter intervention, qualitative feedback was obtained through short interviews at the end of each session, initiated by the question “How was your experience today?” In the final session of the five-week intervention, an extensive text input-based questionnaire was distributed to record the participants’ general assessments. It included exergaming experience, perceived benefits, sentiments towards the single-player and collaborative exergaming modes, usability, and thoughts about the future of the exergame.

#### Physical exertion

As a proxy for assessing each player’s effort associated with physical exertion during the gaming sessions, the total distance travelled by the user’s hands was recorded in accordance with one recent study (Cao et al. 2021). This value was computed by the summation of hand-position offsets (for both hands) through hand tracking provided in the Meta Quest VR headset. The frequency of script updates for tracking hand-position offsets was set at a fixed rate of 50 frames per second.

### Study design

After the iterative codesign and development process, we conducted a five-week study (a total of 99 exergaming sessions), adopting a mixed- methods approach. The 14 participants were divided into two groups of seven and randomly assigned to either the PA or PC exergaming condition. In the first four weeks of the study, we used a between-subjects design. All participants remained in the same groups during the four weeks, and each participant played the exergame twice a week, resulting in a total of 88 sessions for 14 participants. Thereafter, we conducted a cross-sectional study in the last week (with 11 exergaming sessions). Here, the participants played the exergame in counterbalanced order (those playing alone played collaboratively, and vice versa). The aim was to examine the effect of switching the conditions (PC vs PA) on the users’ motivation and physical exertion. The IMI instrument was administered in the first, middle and last sessions of the four-week intervention and one final time during the fifth week. Additionally, we measured the players’ level of physical exertion, carried out field observations, and conducted interviews throughout the intervention. The GEQ was administered at the end of the fourth week, whereas the SSQ, SUS and VEQ were administered at the end of the last session in week five. Since the participants were asked to answer multiple questionnaires during our study, we carefully assessed and monitored the workload on participants, ensuring that we did not overburden them with requests. There were no critical complications in the data-gathering process, possibly due to the participants’ enjoyment of participating in the exergame and its development.

At the beginning of the intervention, a demo of the exergame was provided to all the participants and they were asked to inform the physiotherapists about any discomfort during the sessions. The demo laid out the steps for participants to enter the play area in VR by entering their name, selecting a gender, and choosing their game mode (PA or PC). Afterward, we demonstrated the interface design, interaction paradigms, and reward mechanisms. A familiarization session was then held in which the participants experienced the flow of the exergame to reduce the potential learning effects. They were also asked to fill out the demographic questionnaire. For players engaged in the collaborative mode, both participants were housed in different rooms of the same building, and the exergame session began when both players entered the virtual play area. One participant was teamed with a therapist due to the uneven number of participants in the group. The VR headsets were sanitized at the beginning of each session to minimize the risk of exposure to the coronavirus.

### Statistical analysis

The analysis of the quantitative data was performed using the SPSS statistical package (IBM corporation, SPSS). For all outcome measures included in the statistical analysis, the null hypothesis regarding between-group comparison in the current study states that there is no difference between the two conditions (playing collaboratively versus playing alone). We used independent-sample t-tests for between-group comparison between the two conditions (PC vs PA) when the data are normally distributed and the Mann–Whitney U-test, along with signed intragroup comparisons, when the data are not normally distributed.

We also carried out within-group comparisons based on the cross-sectional study to study the influence of gaming mode when participants playing in the PA condition switched to the PC condition in the last session, and vice versa for the other group. The null hypothesis regarding within-group comparison states that exergaming in counterbalanced order has no effect on the outcome measures. For intragroup comparisons, we conducted the paired-sample t-tests when data were normally distributed and Wilcoxon signed rank tests when data were not normally distributed. Shapiro–Wilk tests were performed to check normality in the dataset. Below, exergaming sessions are denoted as week.session; e.g., W5.1 is the first session of the fifth week. We present the 95% confidence interval ‘*CI*’ of the coefficients and test for significance with ‘*alpha* = 0.05’.

**Fig. 4:**
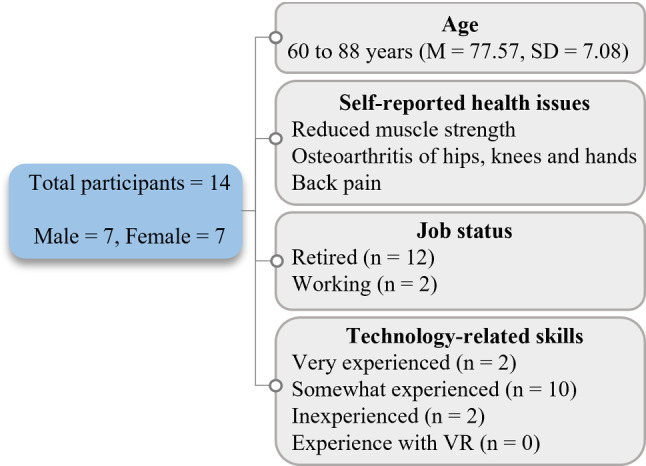
Participant demographics

**Fig. 5:**
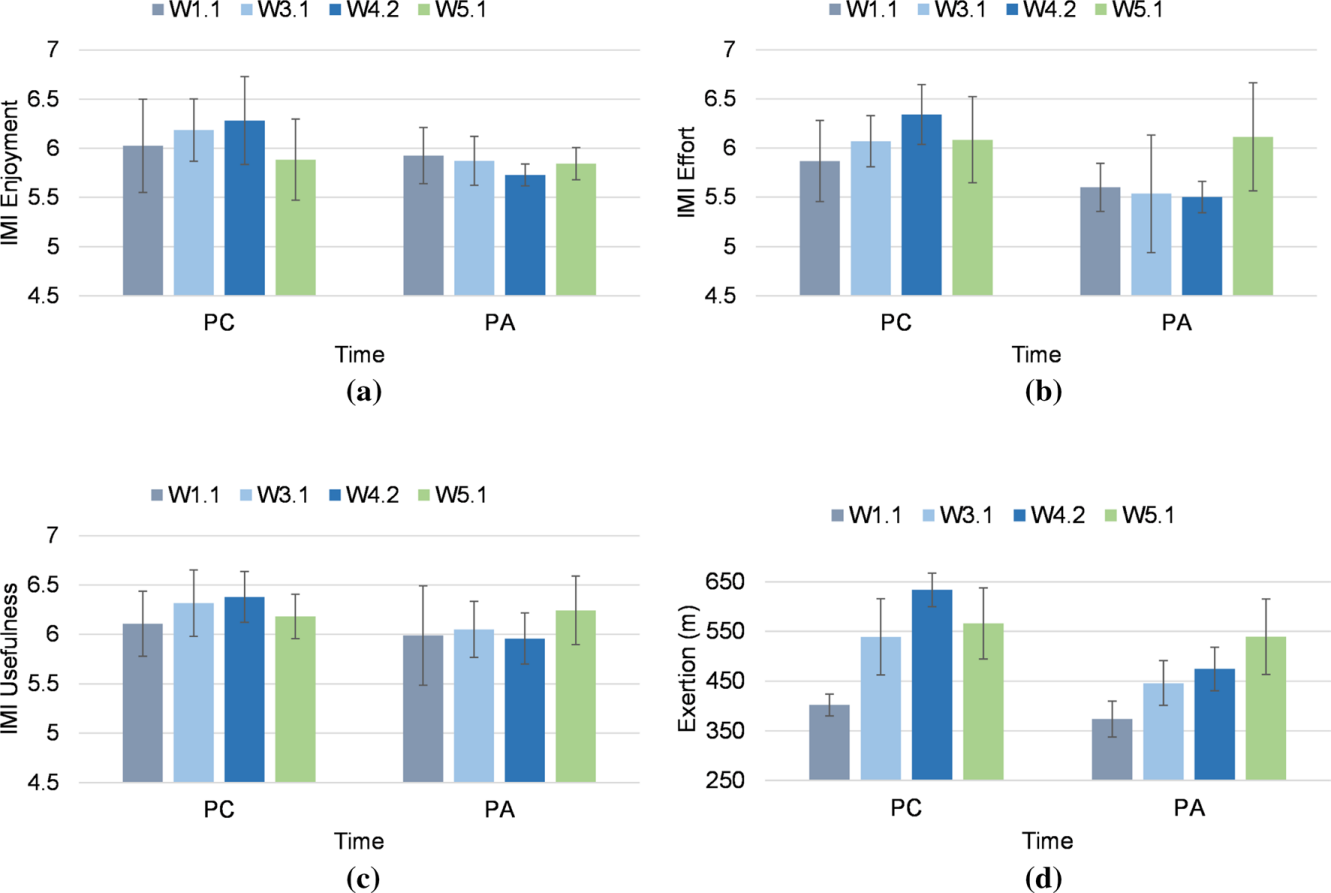
Longitudinal results in PC vs PA for **a** IMI interest/enjoyment, **b** IMI effort/importance, **c** IMI value/usefulness, and **d** physical exertion. (The participants played the exergame in counterbalanced order in W5.1)

## Results

### Participant demographics

A total of 14 elderly individuals (female = 7, male = 7) aged between 60 and 88 years (M = 77.57, SD = 7.08) participated in the five-week intervention. Overall, participants’ demographics are presented in Fig. 4. Two participants still had an active working life, and the other 12 were retired. The participants self-reported health issues included balance problems; reduced muscle strength; osteoarthritis of hips, knees and hands; and back pain. Two participants graded their own technology-related skills as very experienced, ten graded themselves as somewhat experienced, and two considered themselves inexperienced with technology. All the participants were familiar with digital devices for personal or work-related activities, such as smartphones (*n* = 11), simple mobile phones (*n* = 2), mobile tablets (*n* = 8) and laptop or desktop computers (*n* = 8). One participant did not use any digital devices. Digital devices were used for activities such as social networking (*n* = 11), communications with friends or family (*n* = 13), entertainment (*n* = 11), smartphone games (*n* = 4), online shopping (*n* = 1), internet surfing (*n* = 11), work on desktop or laptop computer (*n* = 2), emails (*n* = 4), reading or watching news online (*n* = 4) and checking weather forecasts (*n*=1). None of the participants had previous experience with VR technology.

**Table 1:** Summary of results of intrinsic motivation inventory and physical exertion for each group (mean ± standard deviation)

Variable	W1.1	W3.1	W4.2$^{a}$	W5.1$^{b}$
*Collaborative*				
IMI enjoyment	6.02 ± 0.47	6.18 ± 0.31	6.28 ± 0.44$$**$$	5.88 ± 0.41$$**$$
IMI effort	5.87 ± 0.41	6.07 ± 0.26	6.34 ± 0.31$$***$$	6.08 ± 0.43 $$**$$
IMI usefulness	6.10 ± 0.33	6.31 ± 0.33	6.37 ± 0.25$$**$$	6.18 ± 0.22$$*$$
IMI social con.	5.7 ± 0.8	6.06 ± 0.41	6.46 ± 0.22	−
Exertion (m)	402.24 ± 22.11	538.91 ± 76.92	633.7 ± 33.81$$***$$	565.97 ± 71.9$$*$$
*Alone*				
IMI enjoyment	5.92 ± 0.28	5.87 ± 0.25	5.72 ± 0.11	5.84 ± 0.16$*$
IMI effort	5.60 ± 0.24	5.53 ± 0.59	5.50 ± 0.16	6.11 ± 0.55$$**$$
IMI usefulness	5.98 ± 0.50	6.05 ± 0.28	5.95 ± 0.25	6.24 ± 0.34$*$
IMI social con.	–	–	–	5.2 ± 0.62
Exertion (m)	373.74 ± 36.13	446.04 ± 44.80	474.34 ± 43.41	539.09 ± 76.11$$**$$

$^{a}$Between-group comparison.

$^{b}$Within-group comparison

Significance: $${***}p < 0.003$$; $${**}p < 0.03$$; $${*}p < 0.05$$

### Intrinsic motivation

Shapiro–Wilk tests showed that the data for the IMI subscales collected in each session during the five-week study were normally distributed (*p* > 0.05). Data collected for both groups (PC vs PA) against the IMI subscales are presented in Table 1.

#### Between-group comparison

Independent sample t-tests were performed on the data collected in *W1.1* and *W4.2* to examine whether there were any differences between the collaborative and solo conditions over repeated use. No significant differences between the group baseline scores (*W1.1*) were detected in the IMI subscales, *t* (12) < 1.6, *p* > 0.12. In the subsequent exergaming sessions, the players in the collaborative condition had higher scores in all IMI subscales, as shown in Table 1. Significant differences were found between the two groups in interest/enjoyment scores on the IMI scale in *W4.2*, which suggests that users in the collaborative condition found the exergame more interesting and enjoyable than those playing alone (Fig. 5a, *CI* = [0.14, 0.96], *t*(12) = 2.94, *p* = 0.02). Furthermore, in *W4.2*, users playing collaboratively showed significantly higher IMI effort/importance scores than those playing alone (Fig. 5b, *CI* = [0.35, 1.17], *t*(12) = 4.01, *p* = 0.002). Our interpretation is that collaborative play motivated the participants to make more effort in the exergame than playing alone. Exergaming in collaboration also yielded significantly higher IMI value/usefulness scores than playing alone in *W4.2* (Fig. 5c, *CI* = [0.1, 0.75], *t*(12) = 2.82, *p* = 0.01).

#### Within-group comparison

We used one-tailed paired sample t-tests to examine the effect of switching between the exergaming conditions in *W5.1* on IMI subscale scores based on participants’ responses in *W4.2* and *W5.1*. Comparing these values showed that when those playing alone switched to the collaborative condition in *W5.1*, there was a significant improvement in IMI interest/enjoyment scores (*t*(6) = 1.84, *p* < 0.05) with a medium-sized effect (Cohen’s *d* = 0.69), IMI effort/importance scores (*t*(6) = 2.62, *p* = 0.02) with a large effect (Cohen’s *d* = 0.98), and IMI value/usefulness scores (*t*(6) = 1.96, *p* = 0.04) with a medium-sized effect (Cohen’s *d* = 0.74). Conversely, the data suggest that when players in the collaborative condition switched to playing alone in *W5.1*, their IMI interest/enjoyment scores significantly deteriorated (*t*(6) = −2.42, *p* = 0.02)) with a large effect (Cohen’s *d* = −0.91). Furthermore, there was a reduction in IMI effort/importance scores (*t*(6) = −2.1, *p* = 0.04)) with a medium-sized effect (Cohen’s *d* = −0.79), and a reduction in IMI value/usefulness scores (*t*(6) = − 1.88, *p* < 0.05)) with a medium-sized effect (Cohen’s *d* = −0.71).

Notably, those playing collaboratively had significantly higher IMI social connectedness scores (M: 6.4, SD: 0.2) in *W4.2* compared to their scores in *W1.1* (M: 5.7, SD: 0.8). This suggests that social connectedness and bonding with coplayers over time was important for the players.

### Physical exertion

The data for physical exertion were normally distributed (*p* > 0.05), according to the Shapiro–Wilk tests. Physical exertion data recorded during the five-week study are presented in Table 1.

**Table 2:** Results of the GEQ components for each group (mean ± standard deviation)

Component	PC	PA	*p*-value
*In-game module*			
Competence	3.28 ± 0.21	3.26 ± 0.34	0.55
Sensory and imaginative immersion	3.52 ± 0.48	3.38 ± 0.34	0.56
Flow	3.30 ± 0.36	3.23 ± 0.54	0.79
Tension	0.21 ± 0.14	0.26 ± 0.29	0.89
Challenge	2.5 ± 0.35	2.43 ± 0.73	0.82
Negative affect	0.12 ± 0.07	0.28 ± 0.41	0.78
Positive affect	3.5 ± 0.31	3.43 ± 0.37	1.00
*Postgame module*			
Positive experience	3.38 ± 0.12	3.33 ± 0.55	1.00
Negative experience	0.09 ± 0.15	0.17 ± 0.28	0.70
Tiredness	0.14 ± 0.22	0.28 ± 0.45	0.74
Returning to reality	0.19 ± 0.16	0.38 ± 0.54	0.09

#### Between-group comparison

No significant differences were found between groups in the baselines (*W1.1*) for physical exertion in terms of distance covered by the hands during game play (independent-sample t-tests, *t*(12) = 1.64, *p* = 0.12). Both groups were similarly motivated and made approximately the same effort when playing the exergame. In the ensuing exergaming sessions, participants in the collaborative condition had higher exertion scores than those playing alone (See Table 1). Users playing collaboratively had significantly higher physical exertion than those playing alone in *W4.2* (Fig. 5d, *CI* = [110.38, 208.29], *t*(12) = 7.09, *p* < 0.001). Similarly, based on significant differences observed in IMI effort/importance scores, this suggests that the collaborative mode motivated participants to make more effort while exergaming.

#### Within-group comparison

We used one-tailed, paired sample t-tests to examine the effect of switching between the playing conditions in *W5.1* on physical exertion based on participants’ in-game recorded data during *W4.2* and *W5.1*. This comparison showed that when those playing alone switched to the collaborative condition in *W5.1*, their exertion scores increased (*t*(6) = 2.6, *p* = 0.02), with a large effect size (Cohen’s *d* = 0.98). When participants in the collaborative group played alone in *W5.1*, there was a deterioration in exertion scores (*t*(6) = −1.92, *p* < 0.05) with a medium-sized effect (Cohen’s *d* = −0.72).

### Exergame experience

The game experience questionnaire was administered in the second-to-last session of the five-week intervention (*W4.2*). The purpose was to examine between-subject effects on the overall exergaming experience. Detailed results of the GEQ are presented in Table 2. Shapiro–Wilk tests for normality revealed that data for the items sensory and imaginative immersion, flow, challenge and positive affect were normally distributed. Independent-sample t-tests were therefore performed for these components. In contrast, data for the remaining components were not normally distributed, making them suitable for Mann–Whitney U-tests. The GEQ in-game module examines the user’s experience while playing the game, whereas the postgame module investigates users’ impressions of their physical and mental condition after the game. Statistical tests for each component of the in-game and postgame modules found no significant differences between the two conditions regarding positive and negative exergaming experiences. Those playing in collaborative condition had higher average scores for components assessed as positive and vice versa for negative experiences.

### Simulator sickness

Scores for the SSQ were calculated based on official guidelines (Kennedy et al. 1993). The SSQ was administered in the last week of the five-week study. Scores for the symptoms of nausea (N), oculomotor (O) and disorientation (D) subscales, as well as the total score (TS), are presented as pretest and posttest mean scores (± standard deviation) and change scores (see Table 3).

**Table 3:** SSQ scores (mean ± standard deviation) for all subscales and total scores in pretest, posttest and change scores

Subscale	Pretest	Posttest	Change score
N	4.77 ± 10.66	17.71 ± 15.23	12.94 ± 12.28
O	9.75 ± 10.88	9.75 ± 9.25	0 ± 4.96
D	8.95 ± 14.51	9.94 ± 14.34	0.99 ± 3.58
TS	9.08 ± 11.19	14.43 ± 11.29	5.34 ± 6.74

Notably, a comparatively large difference was observed in self-reports on the item ‘sweating’ on the subscale nausea, indicating that the players were physically engaged in the exergame.

### Virtual embodiment

**Fig. 6:**
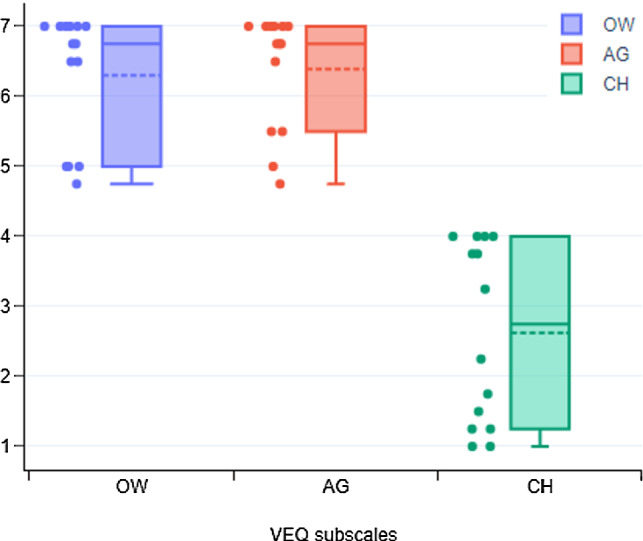
Boxplot for VEQ scores: Ownership (OW), Agency (AG) and Change (CH). The solid line represents median value, the dotted line represents mean value, and the solid round points are independent samples. The inner box shows the interquartile range

An instrument called the Virtual Embodiment Questionnaire was used in week five of the intervention (W5.1). On the factor ownership (OW), which assesses perceptions of ownership of the virtual body, 71.4% of the participants strongly agreed (21.4% agreed and 7.2% somewhat agreed) that they had experienced the virtual body as their body. For the factor agency (AG), which assesses feelings of control over the virtual body, a majority of the participants strongly agreed (71.4%, with 14.3% agreeing and the rest somewhat agreeing 14.3%), that they had control over the virtual body’s movements. The final item, change (CH), assesses perceptions of change in the player’s own body resulting from changes in the virtual body. Half of the players strongly disagreed 50% (with 21.5% disagreeing and 28.5% somewhat disagreeing) that the appearance or form of their own body changed. Mean VEQ scores for all participants across all factors were OW (6.3±0.9), AG (6.4±0.8) and CH (2.6±1.2). Fig. 6 shows the central tendencies of the participants’ responses.

### Usability

The SUS questionnaire was administered in the last week (*W5.1*). It measures the efficiency, effectiveness, and satisfaction of the exergame system in terms of usability. The lowest SUS score calculated from the participants was given by P1 (60), whereas the highest score was given by P3 and P5 (100). The average SUS score was 83.75±13.3. Overall, the players strongly agreed (*n* = 6) or agreed (*n* = 8) that they would like to use the exergame more frequently. The system was not considered unnecessarily complex, and ten participants strongly agreed and four participants agreed that the system was easy to use. Most participants strongly disagreed that they needed detailed technical skills to use the system (*n* = 11). However, two participants agreed that such skills were necessary, while one player remained neutral. Moreover, nine participants strongly agreed that most people would quickly learn the system, four participants agreed, and one participant remained neutral. When the participants were asked about the need for technical support from staff, half strongly disagreed, with four participants disagreeing, two participants agreeing, and one participant strongly agreeing that they needed technical support to use the system. Four participants reported that they felt confident in using the exergame, nine strongly agreed, and one remained neutral.

### Qualitative feedback

Throughout the study, semistructured interviews were carried out with the players to better understand usability and game design issues. To collect qualitative feedback from the participants about their experiences, we administered a semistructured text input questionnaire at the end of the last exergaming session (*W5.1*). We performed a thematic analysis of the qualitative data and present the main findings from these investigations below.

#### General perceptions and future views of the exergame

The participants reported that their overall experience with the exergame was engaging and enjoyable. In the words of one user, “I think the game has contributed to my overall mood as well as physical and social well-being in a positive way” (P1). Another reported, “This game is much better than regular exercise. It would be nice to play it regularly. I forgot that I am practicing. Usually, I don’t do exercise, but this is engaging, and I like it” (P4). Participant seven gave the following statement: “Yes, I enjoyed it. It was a new and exciting experience. It caught my attention” (P7).

None of the participants mentioned particular dislikes, and all reported that they would like to play the exergame more often: “I would like to play this game probably three or four times a week. It has been positive, both socially and physically” (P4). In the words of Participant Seven, “If I had the opportunity, I would play every day. The gaming experience had a very positive effect on my body’s mobility and balance, and it is something I should work with on a daily basis. At the same time, it is also nice and social” (P7). Participant Nine proclaimed, “If the game was available to me, I would have liked to play it several times a week” (P9), and this sentiment was echoed by Participant 10: “I would like to have this game in my daily routine, as it motivates me to do exercise” (P10).

#### Social collaboration appears to motivate exergame play

Overall, the participants reported that the exergame was motivating and enjoyable. It offered a different experience than conventional exercise, with the coplayer mode introducing a novel social and collaborative dimension. In addition to physical health benefits, the intervention offered an opportunity to meet new people and socialize. Most players mentioned that if they had access to a VR headset at home, they would likely play this exergame daily. Participant Six even asked, “Can I have this game at my home?” (P6). Several players stressed the value of the exergame’s social dimensions and how being social through the game motivated gaming activities. This sentiment was articulated by Participant 11 as follows: “I think I would prefer playing with a coplayer. I do not think I would play alone, as I think it would be boring to play alone. In the future, I would prefer having a coplayer for company in the game” (P11). This sentiment was not unanimous, however, and one participant was particularly vocal about preferring to play alone: “I did not like playing with others, as it was disturbing. It took away my focus from the game” (P8).

When asked about their preferences concerning playing alone versus playing with a coplayer, thirteen participants, all except Participant Eight, responded that they would prefer to play the exergame with a coplayer: “I would like to play with a coplayer, as it helps me to do better” (P1). Participant Two: “Playing this game with a teammate is preferable as it helps to be socially active” (P2). Participant Seven: “I feel more excited when playing with another player. It is more rewarding and motivating to play game with a teammate” (P7). Finally, Participant 14: “I would like to play with a teammate; it is fun and social.” Participant Eight elaborated on the preference for playing alone while learning and playing with a coplayer later: “When learning the game, I think playing alone is better, but after I have learned how to start the game and have got more experience, I think I would prefer playing with a coplayer” (P8). While comparing the competitive and collaborative aspects of the exergame, most participants mentioned that they would like to play collaboratively. They did, however, want to know how much they contributed to achieving the in-game rewards: “Nice to have someone to work with and be able to haggle with” and “I like to collaborate with teammates instead of competing. Competition gives a feeling of pressure” (P10).

#### Self-competition as a motivating factor

One prominent aspect of the game that became apparent during the five-week study was self-competition as a motivating factor. Self-competition refers to the desire of performing better and get a higher game score in an upcoming exergaming session than in a previous one. This instilled a sense of mastery and motivated further efforts: “The game challenged and motivated me to try for better performance week by week” (P1); “It is something which I look forward to every week. It has given me a feeling of mastery” (P2); “I like it and I look forward to every game session. I also feel a little uplifted through the week. It was motivating to see my own progress, from not scoring at all to a score of 40 points/fruits” (P5); “Feel a little high and uplifted in mood after playing the game during the week due to performing better than my expectation” (P9); and “I feel happier due to the feeling of mastery in the game” (P13). However, the participants playing individually tended to lose motivation when they did not manage to do better, whereas the coplayers in social collaboration kept each other motivated to do better in upcoming exergaming sessions. This feedback suggests a need to continuously integrate new game challenges and tasks to keep players engaged and motivated over time.

#### Perceived health benefits

In addition to self-reporting positive social experiences from the game, the users positively assessed the exergame’s influence on their own physical health. In the words of Participant One: “I feel the game contributed to my physical well-being in a positive direction” and “I feel more mobile in the upper body” (P1). Participant Eight reported, “The game has contributed to improvements in my activity level” (P8). Some reported experiencing soreness after exercising with the game: “Playing this game has made my arm muscles sore after playing, so I have had a good workout for my arms” (P5) and “I have muscle soreness in both arms and legs a few days after playing the game” (P9). Some participants suffering from arthritis saw the game as beneficial for their condition: “I experience this as good training, better than ’regular’ exercise. I feel that this game does good for hips and back function” (P4); “It feels nice to have a firm ankle during the game play” (P10); “It is a good activity for my fingers (arthrosis), as it helped to increase mobility/reduce rigidity in my finger joints by making me forget about pain in joints and muscles during the game” (P9); and “It is useful for hand movements (osteoarthritis), especially for the thumb and forefinger” (P12).

The participants also self-reported that the game helped with their balance: “It made me physically and socially active during the days I played. I look forward to the game sessions. I felt very tired in the knees after playing. It helped me with the balance in knees throughout the day” (P3); “The game helps me use my body more, which is good for my balance” (P6); “I was not feeling a problem in balancing myself while playing the game, and it was motivating for me that I managed to balance myself better each time I played the game. I feel that my balance can be improved if I keep playing this game” (P8); and “I usually feel unsteady in life but didn’t think or feel poor balance during the game” (P9).

Two self-reports also commented on perceived cognitive consequences of the five-week intervention: “I have improved my eye-hand coordination and now manage to grab the fruits, which I could not manage the first time when I played the game. It improved depth perception in my eyesight. I used to spill a lot of drinks before, but now I spill little” (P5) and “Playing this game probably increases my reflexes and my attention span” (P13). This feedback suggests that VR-based social exergames deserve further exploration as a means of supporting socially active and healthy aging.

#### Physiotherapists’ feedback

We also asked the consulting physiotherapists to play the exergame and interviewed them about their perspectives on its usefulness for the target group. Overall, the feedback from the physiotherapists was positive: “I think that if elderly persons add this type of exercise/game to their normal activities, it would help to improve or maintain their physical function. Primarily, I would say this intervention can help in improving the balance, upper-body mobility (including hands and fingers), stamina in standing and coordination” (Physiotherapist 1). When asked about the social dimension, they saw it as a positive addition to the exergame: “This is of course different from person to person, but my impressions from the user study is that the exergame can improve social life in the way that they feel some sort of unity and a common ground. They have a common goal in the game, and the game also works as a starting point for conversations” (Physiotherapist 2). When asked about its usefulness, they answered that technical innovations such as this particular exergame will be more common in daily life, as younger generations are likely more technology-oriented: “In the present time many elderly persons would struggle to use it on their own because of the technology. But this will probably be easier in the future” (Physiotherapist 1). Moreover, all the physiotherapists believed that exergame technologies could support healthy ageing: “Many of the participants reported that they found the game fun, motivating and they forget about their poor balance/feeling of being insecure - and they ‘forget’ that they are doing exercises. So I think that it could contribute to increased activity and prevent impairment in physical function due to lack of activity in old age, which will contribute to better physical function, reduced risk of falling and reduced occurrence of fractures, and it will eventually reduce the need of health care, which of course will reduce workload at care/rehabilitation wards” (Physiotherapist 1). The physiotherapists recommended that more interesting tasks, such as puzzle games, be added to the exergame to keep elderly participants engaged and motivated over time. They did not recommend using the exergame for patients with severe cerebellar, dizziness, or vestibular disorders.

## Discussion

In this work, we codesigned and developed a social VR-based collaborative exergame for elderly persons. In the past, studies on exergames for physical training and exercise have been based on 2D interfaces (Bezerra et al. 2018). In one study based on a 2D screen-based bowling game, for instance, researchers reported better adherence and enjoyment in users playing together compared to those playing alone (Júnior et al. 2021). However, exergames that explore the space of opportunity in VR remain understudied, especially for elderly users (Campo-Prieto et al. 2021). Consequentially, interest in VR-based physical training for the elderly has increased (Tieri et al. 2018; Campo-Prieto et al. 2021). Most of these VR-applications have been designed as single-player games (Bruun-Pedersen et al. 2016; Stamm et al. 2022; Levac et al. 2019). We therefore lack knowledge about using social VR-based exergames for rehabilitation. Unlike existing VR-based applications and games, this exergame promotes social collaboration among users in a virtual environment to support both social interactions and the users’ physical health. Moreover, unlike existing off-the-shelf exergames, our exergame was codesigned with elderly users and therapists to cater to the particular needs of this user group. Furthermore, the present study takes a holistic approach to users’ interests and experiences as they played collaboratively and alone, with repeated measures of enjoyment and effort in the virtual environment, over a period of five weeks. To the best of our knowledge, such an extensive study on social VR-based collaborative exergame for the elderly has not been done before. Contrary to the reduced motivation that has been observed in competitive games (Song et al. 2013; Pereira et al. 2019), those who played collaboratively during our five-week study significantly improved their motivation, compared to those who played alone. Similarly, a high motivation among elderly participants has been observed in a recent cross-sectional study based on tandem biking in social VR (without gamification) for rehabilitation (Høeg et al. 2021). This study, however, does not consider the impact of social VR over repeated use compared to solo experiences. In contrast, our five-week study offers insights about how social VR impact user motivation and enjoyment compared to solo experience in VR-based exergame over time. More information about key design principles, the benefits of playing alone versus playing collaboratively and the value of VR-based exergames for elderly users are discussed in Section 4.1, 4.2 and 4.3, respectively.

### Key design principles in VR-based exergames for elderly users

Based on our codesign and development process, we suggest that the design of exergames needs to be informed by collaborations with the stakeholders, for instance, elderly users and healthcare professionals in the context of the present exergame. This means properly verifying the key design principles and the chosen game elements with the user group, even if those aspects seem obvious or commonsensical. According to the lessons learned during the design process of this exergame in close collaboration with elderly users and therapists, we report on several design principles, which we believe can be helpful in improving elderly users’ experience with VR-based exergames. We suggest that user interfaces for explorative technologies such as VR must be simple and easy for elderly users to understand to improve the game flow. Moreover, all virtual objects, including interactable game objects and the game environment, should be clearly distinguishable in terms of size or color to avoid ambiguities in the user interface. Moreover, in the context of elderly individuals, these virtual nontangible objects in VR should resemble the same objects in real life to ensure that elderly users can easily recognize them. We also found that animated objects should be avoided in VR-based exergames for elderly users, as their appearance can potentially cause nausea and discomfort. Moreover, people’s reflexes slow with age, negatively affecting the reaction time to stimulus, which was another potential reason to avoid using animated game objects in exergames designed for elderly users. However, after multiple exposures to the exergame and improvement in the functioning of reflexes, the use of animated game objects might be a source of a more engaging VR experience. Crucially, interaction paradigms should be kept simple, preferably by using direct hand manipulation for interaction with virtual objects. Use of external devices, such as hand controllers, for VR-based exergames should be avoided, as this puts unnecessary strain on the users. According to a recent study (Chan et al. 2021), nature-based virtual environments in VR applications can improve mood and reduce stress in senior citizens. Similarly, in our study, the players reported a preference for an immersive virtual environment based on natural scenery. Different colors and sound effects should also be used to provide feedback in response to user interaction with virtual objects in VR. Overall, the participants reported positive experiences with the exergame, which was evidenced by high usability scores and results from the game experience questionnaire. The high usability scores indicate that more usable and sustainable digital solutions to support rehabilitation can be achieved by codesigning and tailoring them to the needs of the target user group. Furthermore, we observed that most of the participants were heavily focused on self-competition and kept track of their own performance throughout the sessions over the five-week period. A sense of accomplishment and mastery followed from improvements in their game play over the time. Hence, continuously integrating new tasks into exergames can potentially keep elderly players engaged over time.

### Playing collaboratively versus playing alone

The results from the five-week study demonstrated that a majority of the participants preferred social interactions in the virtual gaming environment, enjoying virtual coplay rather than solo play. During the exergaming sessions, we observed that the participants were guiding each other’s attention towards certain features of the game play and supporting each other. Hence, we recommend using collaboration-based tasks due to their potential for improving social bonding and support among senior citizens. On the other hand, one of the participants reported that playing with a coplayer negatively affected her learning process in the game. It is therefore possible that playing with a coplayer would be more engaging and effective after the user learns to play the exergame by practicing alone. Our quantitative data also revealed significant differences in motivation and levels of physical exertion between participants who played collaboratively versus those who played alone across the repeated exergaming sessions. The attempts to compete with other player for the end-result have been shown to decrease intrinsic motivation for non-competitive individuals (Song et al. 2013). In our study, the social collaboration appeared to improve effort, motivation, and interest in the exergame. In contrast to those working together, solo players saw a decline in motivation even though self-competition was a motivational factor for both groups.

### Potential benefits of VR-based exergames

Most of the participants positively assessed the exergame’s physical and social value, finding it fun and motivating. Some self-reported that they had perceived improvements in hand strength, balance, and mobility. A few participants also reported that instantiating different game objects at run time could perhaps be helpful to train reaction time and mental focus. Furthermore, one participant wondered whether interaction with interactive objects in VR could be helpful in training depth perception. All the participants reported that the flow of the exergame made them forget about their ailments and even forget that they were, in fact, exercising. Based on the lessons learned during the study, we recommend that exergame activities be based on a sound understanding of the therapeutic needs of the user group and codesigned with health professionals and users. Unlike 2D screen-based exergames, VR makes it possible to stimulate body movements in any possible direction around the user, which can help to integrate various posture transitions into exergames. Moreover, avatar-mediated interaction in VR provides more engaging experience in terms of social connectedness and can improve users’ engagement with game tasks.

### Limitations

One of the main limitations of this study is the limited number of participants (14) and the short span of play time (five weeks), which makes it difficult to generalize the results to a broader population, even though a study (Moore et al. 2011) recommends a minimum of 12 participants to provide valuable information. In addition to the sample size, another potential confounder with respect to our outcome measures is a general lack of control over the daily routines of our participants (outpatients), including their health, medication usage, daily form, mood swings and other sources of variation in the sample. Furthermore, due to COVID-19, we were able to conduct the study over only a five-week period. More rigorous investigation would bring more in-depth knowledge about the effects of the exergame on the relevant outcome measures. While most of the elderly participants in our study were strangers, playing the exergame with close acquaintances, such as friends or family, could also produce interesting results with regard to motivation for playing. Moreover, a design that probes the effects of gender differences on gaming preferences could produce valuable insights, benefiting future game designs. The results of our study might also be specific to the tasks required in our exergame, i.e., reaching the virtual 3D objects (fruits) in virtual space; thus, we advise caution in generalizing the results to other tasks.

## Conclusion and future work

In this article, we reported on a case of codesign and development of a social VR-based collaborative exergame for elderly players. We identified key principles of exergame design based on the lessons learned through this codesign and development process. The game was codesigned to train the full body according to therapeutic goals and to support avatar-mediated social interaction through collaborative game tasks. Important features of the game design include the use of direct hand manipulation as an efficient interaction paradigm, the use of different color schemes and sound effects for delivering feedback about virtual interactions such as grabbing and hovering, the use of a virtual environment representing nature, the value of humanoid avatars, the adaptive positioning of game objects, and social engagement through a team performance-based reward mechanism.

This article presents the findings from a five-week intervention in which the participants played the exergame either collaboratively or alone in terms of self-reported motivation, enjoyment, usefulness, and objective measures of effort. Over a five-week period, playing the exergame collaboratively (compared to playing alone) was significantly more beneficial for motivation, enjoyment, social connectedness, and physical exertion. In terms of user experience, no motion sickness and high scores in comfort, usability, and satisfaction were reported by the participants. Our future research directions include studying the effects of gender and the effects of including new collaborative multiplayer game tasks on motivation and enjoyment. We conclude that it is crucial to consider the design preferences of the user group to maximize motivation and adherence. The exergame design should be tailored to the needs of elderly persons in terms of both usability and therapeutic needs. In contrast to exergaming alone in VR, social VR can be more effective in keeping elderly patients engaged in physical activities. Moreover, social exergames should focus on collaboration rather than competitiveness to avoid any adverse effects. We believe that these insights will be useful in future exergame design processes focusing on similar user groups.

In future research, we would like to explore how social VR-based exergaming sessions based on more than two elderly users affect their motivation, interest and overall gaming experience. Moreover, it would be interesting to gain insight about how exergaming with close acquaintances in social VR affects the motivation and enjoyment of the target group. We would also like to expand the intervention-period to do a more detailed analysis of how social VR-based collaborative exergame affect the health situation of elderly users over time, based on physiological data collected through wearable devices. Future work on social VR-based collaborative exergames should also look into opportunities for codesign of a more varied taskscape for the players and examine the influence of multi-task games versus single-task games on motivation, interest and enjoyment in the target group.

## Data Availability

Available upon request to corresponding author.

## References

[CR1] Babadi SY, Daneshmandi H (2021). Effects of virtual reality versus conventional balance training on balance of the elderly. Exp Gerontol.

[CR2] Baker S, Waycott J, Carrasco R, et al (2021) Avatar-mediated communication in social vr: An in-depth exploration of older adult interaction in an emerging communication platform. In: Proceedings of the 2021 CHI conference on human factors in computing systems, pp 1–13

[CR3] Barathi SC, Finnegan DJ, Farrow M, et al (2018) Interactive feedforward for improving performance and maintaining intrinsic motivation in vr exergaming. In: Proceedings of the 2018 CHI conference on human factors in computing systems, pp 1–14

[CR4] Bezerra IMP, Crocetta TB, Massetti T, et al (2018) Functional performance comparison between real and virtual tasks in older adults: a cross-sectional study. Medicine 97(4) 10.1097/MD.0000000000009612 PMC5794361 29369177

[CR5] Brooke J (1996). Sus-a quick and dirty usability scale. Usability Eval Ind.

[CR6] Bruun-Pedersen JR, Serafin S, Kofoed LB (2016) Restorative virtual environment design for augmenting nursing home rehabilitation. J Virtual Worlds Res 9(3)

[CR7] Cacciata M, Stromberg A, Lee JA (2019). Effect of exergaming on health-related quality of life in older adults: a systematic review. Int J Nursing Stud.

[CR8] Cacioppo JT, Cacioppo S (2014). Older adults reporting social isolation or loneliness show poorer cognitive function 4 years later. Eviden Based Nursing.

[CR9] Campo-Prieto P, Cancela JM, Rodríguez-Fuentes G (2021) Immersive virtual reality as physical therapy in older adults: present or future (systematic review). Virtual Reality, pp 1–17

[CR10] Cao L, Peng C, Dong Y (2021). Ellic’s exercise class: promoting physical activities during exergaming with immersive virtual reality. Virtual Reality.

[CR11] Chan SHM, Qiu L, Esposito G, et al (2021) Nature in virtual reality improves mood and reduces stress: evidence from young adults and senior citizens. Virtual reality, pp 1–16 10.1007/s10055-021-00604-4 PMC8617374 34849087

[CR12] Eckert M, Zarco J, Meneses J, et al (2017) Usage of vr headsets for rehabilitation exergames. In: International conference on bioinformatics and biomedical engineering, Springer, pp 434–442

[CR13] Fraser LE, Mansfield A, Harris LR (2018). The weighting of cues to upright following stroke with and without a history of pushing. Can J Neurol Sci.

[CR14] Gorsic M, Novak D (2016) Design and pilot evaluation of competitive and cooperative exercise games for arm rehabilitation at home. In: 38th Annual international conference of the IEEE engineering in medicine and biology society (EMBC). IEEE, pp 4690–4694 10.1109/EMBC.2016.7591774 PMC5653212 28269319

[CR15] Goršič M, Cikajlo I, Novak D (2017). Competitive and cooperative arm rehabilitation games played by a patient and unimpaired person: effects on motivation and exercise intensity. J Neuroeng Rehabil.

[CR16] Hamari J, Koivisto J (2015). “working out for likes”: an empirical study on social influence in exercise gamification. Comput Human BehaV.

[CR17] Høeg ER, Becermen B, Bruun-Pedersen JR, et al (2019) Co-creating virtual reality applications for motor rehabilitation with physiotherapists. In: Interactivity, game creation, design, learning, and innovation. Springer, pp 379–389

[CR18] Høeg ER, Bruun-Pedersen JR, Cheary S, et al (2021) Buddy biking: a user study on social collaboration in a virtual reality exergame for rehabilitation. Virtual Reality pp 1–18

[CR19] Jaarsma T, Klompstra L, Ben Gal T (2015). Increasing exercise capacity and quality of life of patients with heart failure through wii gaming: the rationale, design and methodology of the hf-wii study; a multicentre randomized controlled trial. Eur J Heart Failure.

[CR20] Jones SA, Alicea SK, Ortega JD (2020). A self-determination theory approach for exercise motivation in rural dwelling older adults. Activ Adap Aging.

[CR21] JustCreate (2021) https://tinyurl.com/2p9vbsvs

[CR22] Júnior JLADS, Biduski D, Bellei EA (2021). A bowling exergame to improve functional capacity in older adults: co-design, development, and testing to compare the progress of playing alone versus playing with peers. JMIR Serious Games.

[CR23] Kappen DL, Mirza-Babaei P, Nacke LE (2019). Older adults’ physical activity and exergames: a systematic review. Int J Human Comput Interaction.

[CR24] Kennedy RS, Lane NE, Berbaum KS (1993). Simulator sickness questionnaire: an enhanced method for quantifying simulator sickness. Int J Aviat psychol.

[CR25] Levac DE, Huber ME, Sternad D (2019). Learning and transfer of complex motor skills in virtual reality: a perspective review. J Neuroeng Rehabil.

[CR26] Li J, Theng YL, Foo S (2020). Play mode effect of exergames on subthreshold depression older adults: a randomized pilot trial. Front Psychol.

[CR27] Lyons EJ, Tate DF, Komoski SE, et al (2012) Novel approaches to obesity prevention: effects of game enjoyment and game type on energy expenditure in active video games 10.1177/193229681200600415 PMC3440155 22920810

[CR28] Maclean N, Pound P, Wolfe C (2000). Qualitative analysis of stroke patients‘ motivation for rehabilitation. Bmj.

[CR29] Marker AM, Staiano AE (2015). Better together: outcomes of cooperation versus competition in social exergaming. Games Health J.

[CR30] Maslow AH (1981) Motivation and personality. Prabhat Prakashan

[CR31] Meekes W, Stanmore EK (2017). Motivational determinants of exergame participation for older people in assisted living facilities: mixed-methods study. J Med Internet Res.

[CR32] Mehra S, Dadema T, Kröse BJ (2016). Attitudes of older adults in a group-based exercise program toward a blended intervention; a focus-group study. Front Psychol.

[CR33] Meldrum D, Glennon A, Herdman S (2012). Virtual reality rehabilitation of balance: assessment of the usability of the nintendo wii® fit plus. Disabil Rehabil Assistive Technol.

[CR34] Meta (2022) https://about.facebook.com/meta/

[CR35] Microsoft (2021a) https://tinyurl.com/h78sw5p2

[CR36] Microsoft (2021b) https://tinyurl.com/2p9d5h9u

[CR37] Moore CG, Carter RE, Nietert PJ (2011). Recommendations for planning pilot studies in clinical and translational research. Clin Transl Sci.

[CR38] Nagano Y, Ishida K, Tani T (2016). Short and long-term effects of exergaming for the elderly. Springerplus.

[CR39] Najafabadi MM, Azad A, Mehdizadeh H (2019). Improvement of upper limb motor control and function after competitive and noncompetitive volleyball exercises in chronic stroke survivors: a randomized clinical trial. Arch Phys Med Rehabil.

[CR40] Nawaz A, Skjæret N, Helbostad JL (2016). Usability and acceptability of balance exergames in older adults: a scoping review. Health Inf J.

[CR41] Nguyen TTH, Ishmatova D, Tapanainen T, et al (2017) Impact of serious games on health and well-being of elderly: a systematic review. In: Proceedings of the 50th Hawaii international conference on system sciences

[CR42] Normal-Inc. (2020) https://normcore.io/

[CR43] Oculus (2020) https://developer.oculus.com/downloads/package/unity-integration/

[CR44] Oculus (2021) https://www.oculus.com/experiences/quest/

[CR45] Peiris CL, Taylor NF, Shields N (2012). Patients value patient-therapist interactions more than the amount or content of therapy during inpatient rehabilitation: a qualitative study. J Physiotherapy.

[CR46] Pereira F, Bermúdez i Badia S, Ornelas R (2019). Impact of game mode in multi-user serious games for upper limb rehabilitation: a within-person randomized trial on engagement and social involvement. J Neuroeng Rehabil.

[CR47] Piech J, Czernicki K (2021). Virtual reality rehabilitation and exergames-physical and psychological impact on fall prevention among the elderly-a literature review. Appl Sci.

[CR48] Poels K, de Kort Y, IJsselsteijn W (2007) D3.3 : Game Experience Questionnaire: development of a self-report measure to assess the psychological impact of digital games. Technische Universiteit Eindhoven

[CR49] Reis E, Postolache G, Teixeira L (2019). Exergames for motor rehabilitation in older adults: an umbrella review. Phys Therapy Rev.

[CR50] Roth D, Latoschik ME (2020). Construction of the virtual embodiment questionnaire (veq). IEEE Trans Vis Comput Graph.

[CR51] Ryan RM, Deci EL (2000). Self-determination theory and the facilitation of intrinsic motivation, social development, and well-being. Am Psychol.

[CR52] Ryan RM, Deci EL (2020). Intrinsic and extrinsic motivation from a self-determination theory perspective: definitions, theory, practices, and future directions. Contemp Edu Psychol.

[CR53] Schroeder SA (2007). We can do better-improving the health of the american people. New Engl J Med.

[CR54] Shah SHH, Hameed IA, Karlsen AST, et al (2022) Towards a social vr-based exergame for elderly users: An exploratory study of acceptance, experiences and design principles. In: International conference on human-computer interaction. Springer, pp 495–504

[CR55] Skjæret N, Nawaz A, Morat T (2016). Exercise and rehabilitation delivered through exergames in older adults: An integrative review of technologies, safety and efficacy. Int J Med Inf.

[CR56] Song H, Kim J, Tenzek KE (2013). The effects of competition and competitiveness upon intrinsic motivation in exergames. Comput Human Behav.

[CR57] Stamm O, Dahms R, Reithinger N, et al (2022) Virtual reality exergame for supplementing multimodal pain therapy in older adults with chronic back pain: a randomized controlled pilot study. Virtual Reality, pp 1–15 10.1007/s10055-022-00629-3 PMC8831688 35194374

[CR58] Tanaka Y, Hirakawa M (2016) Efficient strength training in a virtual world. In: 2016 IEEE international conference on consumer electronics-Taiwan (ICCE-TW), IEEE, pp 1–2

[CR59] Tieri G, Morone G, Paolucci S (2018). Virtual reality in cognitive and motor rehabilitation: facts, fiction and fallacies. Exp Rev Med Devices.

[CR60] Van Santen J, Dröes RM, Holstege M (2018). Effects of exergaming in people with dementia: results of a systematic literature review. J Alzheimer’s Dis.

[CR61] WHO (2021) https://www.who.int/health-topics/ageing#tab=tab_1

[CR62] Wu PT, Wu WL, Chu IH (2015). Energy expenditure and intensity in healthy young adults during exergaming. Am J Health Behav.

[CR63] Wu Y, Lin J, Wu P (2019). Effects of a hybrid intervention combining exergaming and physical therapy among older adults in a long-term care facility. Geriatrics Gerontol Int.

[CR64] Xiao X, Lin Q, Lo WL, et al (2017) Cerebral reorganization in subacute stroke survivors after virtual reality-based training: a preliminary study. Behav Neurol, 2017 10.1155/2017/6261479 PMC5506482 28720981

